# *Chlamydia trachomatis* intra-bacterial and total plasmid copy number in clinical urogenital samples

**DOI:** 10.1038/s41598-020-80645-y

**Published:** 2021-01-08

**Authors:** J. A. M. C. Dirks, K. Janssen, C. J. P. A. Hoebe, T. H. B. Geelen, M. Lucchesi, N. H. T. M. Dukers-Muijrers, P. F. G. Wolffs

**Affiliations:** 1 Department of Medical Microbiology, Maastricht University Medical Center, Care and Public Health Research Institute (Caphri), Maastricht, The Netherlands; 2 Department of Sexual Health, Infectious Diseases and Environmental Health, Public Health Service South Limburg, Geleen, The Netherlands

**Keywords:** Bacterial infection, Urogenital diseases

## Abstract

*Chlamydia trachomatis* (CT) increases its plasmid numbers when stressed, as occurs in clinical trachoma samples. Most CT tests target the plasmid to increase the test sensitivity, but some only target the chromosome. We investigated clinical urogenital samples for total plasmid copy numbers to assess its diagnostic value and intra-bacterial plasmid copy numbers to assess its natural variation. Both plasmid and chromosome copies were quantified using qPCR, and the plasmid:chromosome ratio (PCr) calculated in two cohorts: (1) 383 urogenital samples for the total PCR (tPCr), and (2) 42 vaginal swabs, with one half treated with propium-monoazide (PMA) to prevent the quantification of extracellular DNA and the other half untreated to allow for both tPCr and intra-bacterial PCr (iPCr) quantification. Mann–Whitney U tests compared PCr between samples, in relation to age and gender. Cohort 1: tPCr varied greatly (1–677, median 16). Median tPCr was significantly higher in urines than vaginal swabs (32 vs. 11, p < 0.001). Cohort 2: iPCr was more stable than tPCr (range 0.1–3 vs. 1–11). To conclude, tPCr in urogenital samples was much more variable than previously described. Transport time and temperature influences DNA degradation, impacting chromosomal DNA more than plasmids and urine more than vaginal samples. Data supports a plasmid target in CT screening assays to increase clinical sensitivity.

## Introduction

*Chlamydia trachomatis* (CT) is the most common bacterial sexually transmitted infection (STI) worldwide, with over 100 million people affected^1^. The clinical picture is highly variable, ranging from virtually no symptoms to pelvic inflammatory disease and infertility^2^. The bacterium contains a highly conserved small plasmid, an extra-chromosomal genetic element capable of self-replication, that confers some type of benefit to bacterial survival^3^. The CT plasmid is multifunctional, with effects on both the regulation of chromosomal gene expression and virulence^4,5^. Plasmidless strains are rare and exhibit a diminished virulence^6–8^. Generally, the number of CT plasmids in laboratory strains is stable and ranges from 1 to 10 plasmid per bacterium^9–11^. However, CT can increase its plasmid numbers when stressed^9^, as is likely to happen in the human body^12^. In that light, it has been demonstrated that the plasmid copy number is more variable in clinical trachoma samples, with 1–18 plasmid per bacterium^13^. It is currently unknown if a similar scenario occurs in clinical genital samples. To study this further, we assessed plasmid copy numbers in clinical urogenital samples using quantitative PCR (qPCR) and viability PCR (V-PCR), and related this to chromosome copies. QPCR is a sensitive method to quantify plasmid and chromosomal copy numbers in a sample, but cannot distinguish between extra-bacterial and intra-bacterial plasmids. To make this distinction, we used V-PCR, which has been extensively described elsewhere^14–16^. In short, it uses propidium monoazide (PMAxx), which binds extra-bacterial DNA (including plasmids), ensuring that all amplified DNA is from intact bacteria. The combination of these two methods will provide additional insights in plasmid copy numbers in clinical urogenital samples to provide further support to current guidelines.

## Results

### Patient population

#### Cohort 1

A total of 497 urogenital samples were included from unique patients. In 385 (77.5%) samples both the plasmid and chromosome concentration could be quantified (0.2% no plasmid detected, 15.7% no chromosomal DNA detected, 6.6% neither plasmid nor chromosome detected). One patient had more chromosomes than plasmids present in her sample, a deviant result, which we could not confirm as there was no sample left. We have therefore excluded this patient from the results. Patient data were missing in 2 cases, resulting in 382 samples from unique patients. The majority of patients was female (61%, n = 233), with a median age of 22 years [IQR 20–24].

#### Cohort 2

A total of 69 self-collected vaginal swabs were included. Of these samples, 55 (79.7%) generated valid results without the use of PMAxx (6 no chromosomal DNA, 0 no plasmid detected), and 46 (66.7%) with the use of PMAxx (15 no chromosomal DNA, 1 no plasmid or chromosomal DNA detected). Both tPCR (without PMAxx) and iPCr (with PMAxx) were available for 42 samples. The median age was 22 years [IQR 20–24], identical to that of cohort 1.

### Plasmid:chromosome ratio

#### Cohort 1

**Figure 1:**
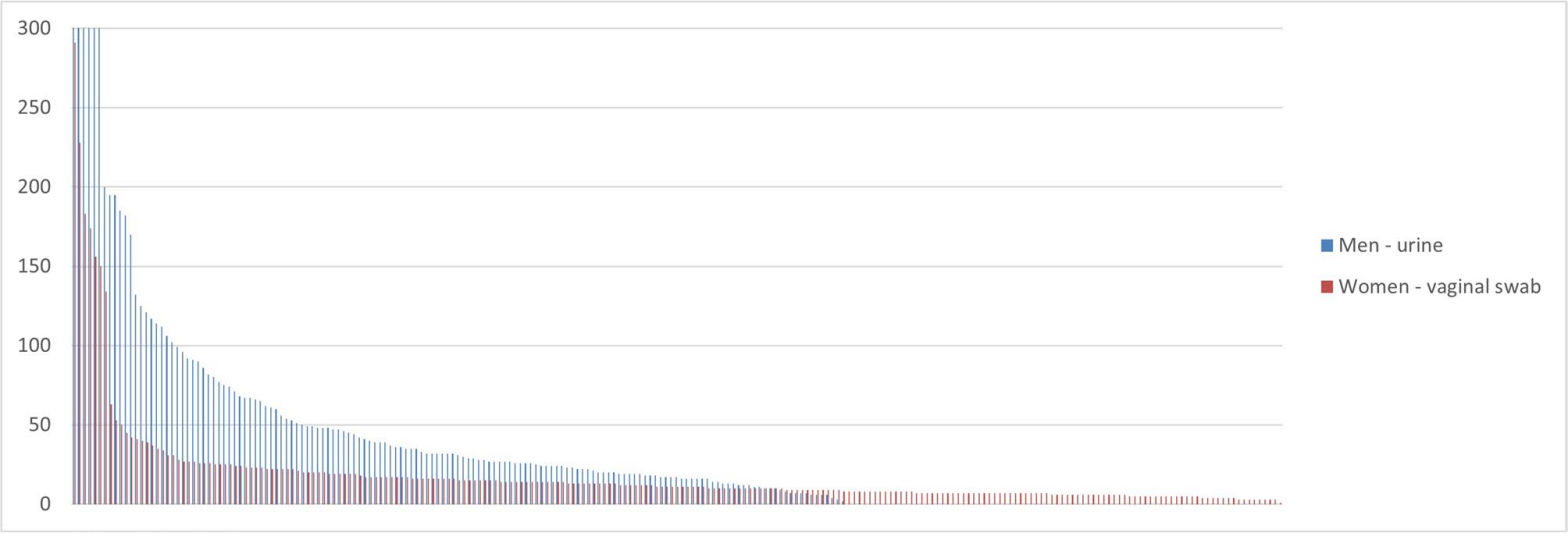
Frequency distribution of the tPCr in urine samples (blue) and vaginal samples (red) from patients from cohort 1. Each bar indicates an individual patient.

The tPCr varied from 1 to 677. The majority of samples (217; 57%) contained ≤ 18 plasmids per chromosome, comparable to the number found in clinical trachoma samples. Of all samples, 33.2% had tPCr ≤ 10, 48.4% a tPCr ≤ 15 and 75.1% a tPCr ≤ 30. The distribution of the tPCr can be seen in Fig. 1. The tPCr distribution was significantly different between vaginal swabs and urine, with a median tPCr of 11 [IQR 7–17] for vaginal swabs and 31 [IQR 18–63] for urine (p < 0.001).

Age was related to the tPCr, but only for men (p = 0.046), and not for women (p = 0.097). Men of 21 years or younger had a median tPCr of 41 [IQR 21–99], while older men had a median tPCr of 29 [IQR 17–54].

#### Cohort 2

**Figure 2:**
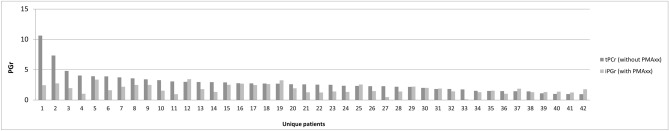
Frequency distribution of the tPCr in vaginal samples from patients from cohort 2. Dark grey bars show the tPCr in samples not treated with PMAxx, this includes both intra-bacterial and extra-bacterial plasmids and chromosome copies. Light grey bars show the intra-bacterial PCr in samples treated with PMAxx. PMAxx prevents the amplification of extra-bacterial plasmid and chromosomal DNA. Each number indicates a unique patient.

The tPCr ranged from 1 to 11 (in samples without PMAxx), with a median of 3 [IQR 2–3], as can been seen in Fig. 2. The iPCr ranged from 0.1 to 3 (in PMAxx treated samples), with a median of 2 [IQR 1–2], see Fig. 2. Age was unrelated to the tPCr (p = 0.5).

## Discussion

This is the first study to investigate the plasmid variability in self-collected urogenital samples from patients visiting an STI-clinic, with the possibility to assess the total and intra-bacterial plasmid fraction. The iPCr showed a narrow range from 0.1 to 3. The tPCr, however, was much more variable. Approximately 75% of samples had a tPCr ≤ 30, but in some samples a tPCr > 100 was found. Furthermore, the tPCr was higher in urine samples than in vaginal swabs.

The tPCr was much more variable than previously described from culture studies^9–11^ and in clinical trachoma samples^13^. Only 33% of all samples had a tPCr ≤ 10, which is generally found in vitro studies. The largest tPCr variation was seen in male urine samples. We believe that this variation is largely due to DNA degradation, which differs between urine samples and vaginal samples due to the sampling/storage and method of transport. After sampling, vaginal swabs were stored in Roche PCR medium (a nucleic acid stabilising transport and storage medium) during transport, which prevents DNA degradation, while urines were not. Previous studies have demonstrated that the small chlamydial plasmid is stable and can be detected for up to 2 years after sampling^17,18^. However, we believe that the chromosomal DNA is not as stable at room temperature and degraded more rapidly than the CT plasmid, especially without the use of a storage buffer. We have demonstrated this in a small study (30 samples, unpublished data). This phenomenon has also been demonstrated for *E. coli*^19^. The tPCr differences between the two cohorts further illustrates the effect of transport-time and storage-temperature on CT degradation. The samples from cohort 1 were sent by mail, whereas samples from cohort 2 had a much shorter time between sampling and processing, were transported on dry ice, and thus a smaller variation in tPCr.

These findings demonstrate an additional reason for the use of the CT plasmid in commercial assays^20^. It has been known that new genetic variants might be missed by single-target assay, as demonstrated by the Swedish new variant CT^21,22^, and since then it is recommended to use dual-target assays^23–25^. Yet, of the five FDA approved and commercially available NAATS (Roche Diagnostics, Becton Dickinson and Company, Hologic/Gen-Probe Inc., Abbott Laboratories, and Cepheid), one detects only a chromosomal target, two only plasmid targets, one detects rRNA and only one detects both^26^. Our results further support the use of dual target assays, as assays targeting only a single chromosomal target lack clinical sensitivity, especially when a cold transport chain cannot be maintained, or when the time between sampling and testing exceeds one day (both are relevant for home-sampling). The exact number of plasmids in a clinical urogenital sample was thus far unknown, but the high tPCr numbers demonstrated in cohort 1 make them an even more sensible target for CT detection than was previously thought.

The iPCr was almost exactly as described in previous literature using culture studies^9–11^, with a maximum of 10 plasmids per chromosome. It has been hypothesized that CT can increase its PCr in response to stressors^9^, as might happen in the human body^12^. Other theories suggest that the CT inclusion is a rather 'mild' environment, in which CT experiences minimal stressors^27^. Our results are more in line with this latter theory, as we detected low iPCr numbers in all samples.

Throughout this article, we refer to the intra-bacterial plasmid:genome ratio in cohort 2, as we assume that freezing the samples lyses most, if not all, human cells, while leaving CT bacteria intact. Chlamydia occurs in two developmental forms. One is the extracellular infectious elementary body (EB), which is highly stable due to extensive disulfide cross linking of its outer membrane. The second is the noninfectious intracellular reticulate body (RB) which is fragile, due to a lack of disulfide cross linking of the outer membrane and traditional peptidoglycan. It could be that the RB’s are lysed together with the human cells while freezing, and the iPGr is thus only a reflection of the PGr in EB’s. However, the minimal difference in both sample-groups in cohort 2 makes this unlikely, as one would expect greater differences if that was the case. This further supports the theory that the differences in the cohorts are caused by transport conditions more than a PGr difference between EB’s and RB’s.

Our study has some limitations, firstly the lower sensitivity of our in-house quantification assay, compared to the commercial assay used for the initial CT screening. This assay detects both plasmid and chromosome, and the presence of either results in a positive result. Our in-house quantification assay is less sensitive than this commercial assay, which is why we could only include ~ 78% of the positive samples, both in the stored samples of cohort 1, as in the fresh samples from cohort 2. Furthermore, the chromosomal *OmpA*-pcr has a lower sensitivity than the plasmid PCR, resulting in a fraction of samples excluded with a bacterial load too low for quantification. Next, for the first cohort we used stored samples, which could theoretically impact the plasmid and chromosome quantity. We performed separate analyses (not included in the manuscript) that demonstrate that the impact of (degradation due to) storage was minimal over time. The analyses include a repeat of a subset of the samples, which showed the same tPCr. This is further supported by literature^18^. Also, time-analyses showed that the samples from 2010 did not yield significantly different results than the samples from 2011 or 2012. And lastly, no men were included in the study using PMAxx, which would have shed more light on the intracellular PCr in urine samples.

In short, we demonstrated that self-collected urogenital samples from patients visiting the STI-clinic show a higher variability of the chlamydial plasmid in urogenital samples than was previously thought. This study also demonstrated that the majority of these plasmids are extracellular, and that the PCr inside CT bacteria is quite stable during infection. With the high plasmid numbers demonstrated in all samples, it seems pertinent that all commercial assays should target the plasmid, in addition to the chromosome, to significantly increase the sensitivity of their test, yet ensure that possible mutants are detected.

## Methods

### Patient population and sample collection

**Figure 3:**
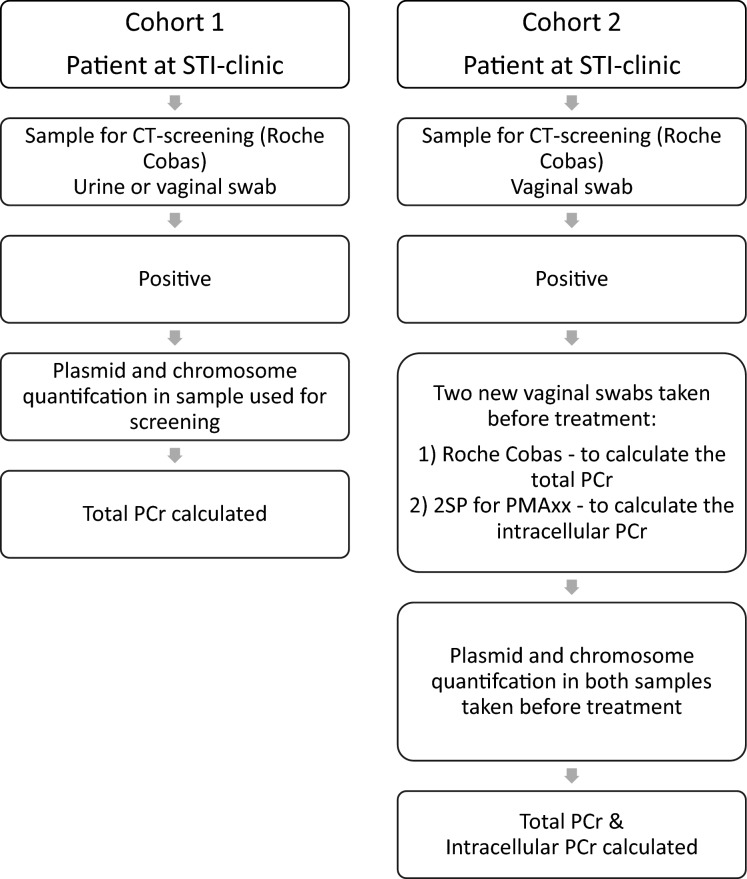
Flow-chart of patient inclusion in cohort 1 and cohort 2.

Samples from two convenience cohorts from our STI-clinic in South Limburg, the Netherlands were included (for a flow-chart of patient inclusion see Fig. 3):

1. *Cohort 1 (qPCR)* CT positive vaginal swabs and male urines were retrospectively included (November 2010—May 2012). Samples were tested for CT with either COBAS Amplicor (Roche Diagnostics, Basel, Switzerland) or COBAS 4800 (Roche Diagnostics) as per manufacturer’s protocol. After sampling, specimens (urine without transport buffer and vaginal-swabs in transport buffer) were stored at 4 °C and transported by cooled transport to the Medical Microbiology Laboratory of the Maastricht University Medical Center (MUMC +) within 24 h, and processed as soon as possible. Exclusion criteria were HIV-positivity or antibiotic use in the previous month. Retrospective review of questionnaires and clinical data provided information about age and gender. If a patient was tested more than once during the study period, only the first sample was included. The Maastricht University Medical Centre Medical Ethics Committee approved this study (METC azM/UM nr.13-4-026). Patients were part of a previously described study assessing the bacterial CT load in urogenital samples^28^. All experiments were performed in accordance with relevant guidelines and regulations (including informed consent from all participants).
2. *Cohort 2 (V-PCR)* Female patients of 18 years or older were prospectively included between November 2015 and April 2016. When the vaginal sample tested CT positive at the initial (screening) visit, patients were asked to provide two self-collected vaginal swabs at the second visit before receiving treatment (pre-treatment samples). The screening sample and one of the pre-treatment samples were both tested for CT with COBAS 4800 (Roche Diagnostics, Basel, Switzerland) as per manufacturer’s protocol. The other pre-treatment sample was placed in a tube containing 2-sucrose-phosphate CT transport medium (2SP) and immediately stored at 4 °C, to use for V-PCR. Both pre-treatment samples were transported within 12 h to the Medical Microbiology Laboratory of the Maastricht University Medical Center (MUMC +) by cooled transport on dry ice (-80 °C). This protocol results in a maximum retention of viable bacteria, as shown by CT culture (data not shown). Exclusion criteria were pregnancy, antibiotic use in the previous month, and having a concurrent infection with *Neisseria gonorrhoeae*. The Maastricht University Medical Centre Medical Ethics Committee approved this study (METC azM/UM nr. 10-4-66). Details of the study are described elsewhere^29^. All experiments were performed in accordance with relevant guidelines and regulations (including informed consent from all participants).

### Viability PCR–PMA treatment

Prior to DNA purification, 2SP samples were split into two aliquots and one aliquot was treated with the membrane impermeable DNA binding dye PMAxx (Biotium, Inc., Hayward, California) as described before^29^. PMAxx stock solution was added to a final concentration of 50 µM per sample. Following an incubation period of 10 min in the dark at 4 °C, samples were placed in the PMA-Lite LED Photolysis Device for 10 min to ensure irreversible binding of PMAxx to DNA from dead bacteria.

### CT load quantification

From 200 µl of each sample total nucleic acids were isolated using the QIAamp DNA Mini Kit (Qiagen, Hilden, Germany) as per manufacturer’s protocol, and eluted in 120 µl elution buffer. The eluate was stored at − 20 °C and thawed once for quantification.

CT load was quantified using qPCR targeting a chromosomal (*OmpA-*gene) and plasmid target, as described elsewhere^28,30^. For cohort 1, PCR amplification was performed in a total volume of 25 µl, consisting of 10 µl DNA and 15 µl reaction mixture containing 12.5 µl Absolute qPCR Rox Mastermix (Thermo Scientific, Waltham, USA) and 2.5 µl primer/probe mix consisting of 840 nM forward and reverse primer and 100 nM probe. For cohort 2, PCR amplification was performed in a total volume of 50 µl. The amplification reaction consisted of 15 min of initial activation at 95 °C, followed by 42 cycles of 95 °C for 15 s and 60 °C for 60 s.

Cycle threshold (Cq)-values were entered into the master curve (calculated from over 10 dilution series) to calculate the CT load (log_10_ CT/ml). Both chromosomal load and plasmid load per sample were then exponentially transformed to achieve an absolute value. The absolute number of plasmids was divided by the absolute number of chromosomal copies, to calculate the number of plasmids per bacterium. This will be referred to as the plasmid:chromosome ratio (tPCr) in the remainder of the article. The tPCr of samples not treated with PMAxx will be referred to as the tPCr, and of samples treated with PMAxx as the intra-bacterial PCr (iPCr).

### Statistical analysis

tPCr was not normally distributed. Mann–Whitney U tests were used for data analyses concerning gender and age. Age was divided into two groups (≤ 21 years and > 21 years). Results were considered statistically significant at p ≤ 0.05. All statistical tests were performed using SPSS (IBM Corp. IBM SPSS Statistics for Windows, Version 24.0. Armonk, NY, USA).

## Data Availability

All data generated or analyzed during this study are included in this published article (and its Supplementary Information files).
